# Cowpea Constraints and Breeding in Europe

**DOI:** 10.3390/plants12061339

**Published:** 2023-03-16

**Authors:** Efstathia Lazaridi, Penelope J. Bebeli

**Affiliations:** Laboratory of Plant Breeding and Biometry, Department of Crop Science, Agricultural University of Athens, Iera Odos 75, 11855 Athens, Greece; e.lazaridi@aua.gr

**Keywords:** abiotic stresses, biotic stresses, breeding methods, crop wild relatives, landraces, nutritional composition, yield

## Abstract

Cowpea (*Vigna unguiculata* (L.) Walp.) is a legume with a constant rate of cultivation in Southern European countries. Consumer demand for cowpea worldwide is rising due to its nutritional content, while Europe is constantly attempting to reduce the deficit in the production of pulses and invest in new, healthy food market products. Although the climatic conditions that prevail in Europe are not so harsh in terms of heat and drought as in the tropical climates where cowpea is mainly cultivated, cowpea confronts with a plethora of abiotic and biotic stresses and yield-limiting factors in Southern European countries. In this paper, we summarize the main constraints for cowpea cultivation in Europe and the breeding methods that have been or can be used. A special mention is made of the availability plant genetic resources (PGRs) and their potential for breeding purposes, aiming to promote more sustainable cropping systems as climatic shifts become more frequent and fiercer, and environmental degradation expands worldwide.

## 1. Introduction

Cowpea (*Vigna unguiculata* (L.) Walp.) (2n = 2x = 22) is an important legume species, both for its consumption as food and as animal feed worldwide, especially in semi-arid tropical and desert regions [1,2]. It is an excellent source of vitamins, antioxidants, fiber, trace elements and other nutrients [2,3] and plays an important role in malnutrition avoidance in the least developed countries (LDCs) where it is mainly cultivated [4,5]. Almost all its above-ground plant parts are consumed [6]. In addition to its mature dry seeds, its leaves, green pods and green seeds are consumed in various countries [5,7,8,9,10]. It is also used for flour [3,11,12,13], as its seeds contain a high protein content (23–32%) compared to many other legume species [14,15].

In addition to its important nutritional potential, its short biological cycle makes this crop ideal for participation in organic farming systems due to its high rates of nitrogen fixation, phosphorus use efficiency and regrowth capacity [16,17]. Like other leguminous crops, it enhances soil improvement due to its ability to coexist with several *Rhizobium* bacteria species [18,19], resulting in the enrichment of the soil with nitrogen [20,21]. Cowpea can also improve soil structure by forming a deep root system and reduce soil erosion due to the extensive coverage that the plants provide [22].

## 2. Botanical Taxonomy

The genus *Vigna* belongs to the legume family and currently includes approximately two hundred species [23], among them ten cultivated species, such as cowpea (*Vigna unguiculata* (L.) Walp.) and green mung bean (*Vigna radiata* (L.) Wilczek) [24]. The genus was established in 1824 by the botanist Savi, who named it in honor of Domenico Vigna, a professor of botany in Pisa [25]. Before the creation of the genus *Vigna*, the species classified in it were previously classified either into the genus *Dolichos* or into the genus *Phaseolus* [26].

Initially, the genus *Vigna* included very few species, *Vigna glabra* Savi and *V. villosa* Savi, which today both belong to the species *V. luteola* (Jacq.) Benth. It was not until 1842 that Walpers transferred cowpea (*Vigna unguiculata* (L.) Walp.) to the specific genus [25]. The species of the genus are now grouped into six subgenera (*Vigna, Ceratotropis, Plectotropis, Sigmoidotropis, Lasiosporon* and *Haydonia*) [27,28] from eight originally [29], while more recent studies through molecular phylogenetic analyses recommended the removal of the subgenus *Sigmoidotropis* [30,31]. The subgenus *Vigna* is further divided into six sections; *Catiang*, *Comosae* and *Macrodontae* each includes two species, *Liebrechtsia* includes one species, *Reticulatae* includes nine species and *Vigna* includes twenty species [24].

Cowpea is classified into the *Catiang* section of the subgenus *Vigna* [32]. The species before its current name had received many names during taxonomic efforts, and this is the reason why it has several synonyms such as *Dolichos biflorus* L., *Dolichos melanopthalmus* DC., *Dolichos unguiculatus* L., *Vigna catjang* (Burm. f.) Walp., *Vigna sinensis* (L.) Savi ex Hausskn. and *Vigna unguiculata* (L.) Walp. ssp. *dekindtiana* sensu Verdc. [33]. The cultivated types of the species (*Vigna unguiculata* ssp. *unguiculata* var. *unguiculata*) are further categorized into four cultivated groups (cultivar groups, cv.-gr.): sesquipedalis, textilis, biflora and unguiculata [26,34], while Pasquet [35] proposed the introduction of one more cultivated group, melanophthalmus, separating types with black eye and wrinkled seed coat morphotypes from the group cv.-gr. unguiculata. In many cases, these cultivar groups are considered by some researchers as separate subspecies and not as cultivar groups of a specific subspecies [36]. Furthermore, the *Vigna* subgenus includes ten wild subspecies [37], as well as wild forms within the subspecies *V. unguiculata* ssp. *unguiculata*, e.g., *Vigna unguiculata* ssp. *unguiculata* var. *spontanea* [38,39].

## 3. Origin and Spread

Cowpea (*Vigna unguiculata* (L.) Walp.) was domesticated in Africa before 1500 B.C. [40]. Southeast Africa or West and Central Africa [25,41,42,43,44] are suggested as the primary center of the species origin. The existence of a parallel double center of cowpea domestication was mentioned lately [45]. The analysis of 1200 single nucleotide polymorphisms (SNPs) led to the formation of two distinct groups of local populations, one similar to wild species originating from Southeast Africa and one to wild species originating from West and Central Africa [46]. In fact, local varieties from Europe appeared to be more similar to the group originating from West and Central Africa, and local varieties from Asia and America to the group originating from Southeast Africa. Landraces originating from Southeast Africa also showed greater interpopulation diversity compared to West and Central Africa [46]. Xu et al. [36], analyzing the genetic similarity of 95 entries of yardlong bean (*Vigna unguiculata* cv.-gr. sesquipedalis), categorized the accessions into two separate groups according to their pod length and growth habit. In addition, Herniter et al. [47], presenting linguistic data for cowpea in the two African regions (Southeast Africa/West and Central Africa) seem to converge on the existence of two parallel centers of origin.

It seems that cowpea was first introduced into Asia during the Neolithic period, around 1500 B.C. [48,49], and in India, a secondary center of origin for the species was formed. Upon entering Asia, cowpea encountered different climatic conditions, and after selection for fresh pod consumption, the cultivated group *V. unguiculata* ssp. *unguiculata* cv.-gr. sesquipedalis was formed [45,46]. This cultivated group was later introduced into the European continent. Asia is also a primary center of origin for many *Vigna* species, such as *V. radiata* and *V. mungo*. Their wild ancestors are also abundant in the area, such as *V. unguiculata* ssp. *unguiculata* var. *sublobata* and *V. unguiculata* ssp. *unguiculata* var. *silvestris* [50]. Along with the spread of cultivar group *V. unguiculata* ssp. *unguiculata* cv.-gr. sesquipedalis in the European area, the spread of the African cultivated *Vigna* (*Vigna unguiculata* ssp. *unguiculata* cv.-gr. unguiculata) probably also took place [51]. As testified by the texts of Theophrastus, cultivation of the “bean” was familiar to the ancient Greeks in 300 B.C. [52]. The two cultivated groups later spread from Europe to South America during the 17th century A.D. [51], and then to the USA during the 18th century A.D. [53].

Today, cowpea is cultivated in areas of Southern Europe such as Greece, Italy, Spain, Cyprus, Croatia, Portugal and Serbia, Bosnia and Herzegovina, and North Macedonia [54,55,56,57,58,59], but is less widespread in Central Europe [52]. Cultivated areas for cowpea dry beans production are also reported in Slovenia and Hungary [58]. In total, cowpea corresponds to the 0.3% of pulses production in Europe and the 0.3% of world cowpea production for dry seed, reaching up to 23,825.22 ton in 2021 [58,60]. However, not many data are available regarding cowpea production for fresh pod production despite that its production was enhanced in Southern European countries lately [10,61].

## 4. Breeding Methods

Maximizing seed yield is the main breeding objective for cowpea [49,62]. Yield is also considered by farmers as the most desirable trait, taking also into consideration its limiting factors [63,64,65]. Classical breeding methods that are used mainly for cv.-gr. unguiculata are pedigree selection, mixed-population selection and single-seed-derived selection [5,66,67,68]. Selection of pure lines has also been used mainly to form parental lines, as cowpea is a species characterized by a narrow genetic base [69,70] and this method does not have the ability to produce new available genetic variability. As for the cultivated group cv.-gr. sesquipedalis, many cultivars have been created through pure-line selection [67]. To introduce a desired trait into an already adapted parent, backcrosses are usually applied [71,72,73]. The preferred breeding method is directly dependent on the total variability available, the heritability (*H* or *h^2^*) of the trait of interest and the selective pressure exerted.

The heritability of various traits associated with cowpea fresh pod and seed yield differs depending on the available variability in the evaluated populations and the environmental conditions and is presented in Table 1 and Table 2 for cv-gr. unguiculata and cv.-gr. sesquipedalis, respectively. Comparing the two main cultivated cowpea cultivar groups (cv-gr unguiculata and cv-gr sesquipedalis), one observes that more traits, such as plant height and fresh pod yield, with high broad sense heritability (*H* > 70%) and significant genetic advance are presented in the cv-gr sesquipedalis cultivar group.

Use of molecular markers aiming to assist classical breeding methods is also common, thus reducing the time required to make a new variety available. Molecular markers are used either by assisting classical methods with trait selection (MAS), or by assisting the detection of a specific trait in backcrossing (MABC), or by assisted recurrent selection (MARS) [106]. In recent years, genome sequencing resulted in significant progress through genetic quantitative trait loci (QTLs) identification, using either linkage mapping or association mapping and revealing the linkage of these genomic regions to phenotype [107]. As a reference the genome of IT97K-499-35 breeding line from Nigeria was sequenced [108], with an assembled genome size of 519.44 Mb [109].

Genome-wide association studies (GWAS) were therefore helped in this direction, through SNPs and candidate genes identification and their correspondence to desirable plant traits such as flowering time and pod indehiscence, and tolerance to biotic and abiotic stresses [46,110,111,112,113,114,115,116,117,118,119,120,121,122]. Cowpea genetic similarities with other widely studied legumes such as soybean (*Glycine max* (L.) Merr.), alfalfa (*Medicago truncatula* Gaertn.) and common bean (*Phaseolus vulgaris* L.) have contributed to better understanding, mapping and confirmation of cowpea QTLs in relation to expressed characteristics [108,123,124,125] (Table 3).

Resistance to abiotic factors, such as drought and high temperatures [123,134], and biotic factors such as resistance to the fungus *Macrophomina phaseolina* [135], aphids [136], nematodes [137] and thrips [138], have also been facilitated through finding the corresponding QTLs. To investigate quantitative trait loci in cowpea, either generation F_3_ populations (F_2:3_) or recombinant homozygous lines of RILs are established [131,137,139,140,141].

Other breeding methods are also used to increase the available cowpea genetic diversity [142]. Induced mutagenesis offers the possibility of generating variability in a shorter period compared to classical breeding methods [143]. Cowpea traits that have been shown to be positively affected by induced mutagenesis are plant height, number of pods per plant, pod length, number of seeds per pod, seed yield per plant and seed protein content [144,145]. Gamma radiation (γ) is the most frequently used method with the highest success rates [142,145,146,147,148], while the use of chemical induction with ethyl methane sulfonate ester (EMS) in concentrations of 0.25% to 0.40% also has significant success rates [146,149,150,151]. The use of sodium azide (NaN_3_) in low concentrations also resulted in encouraging results [148,149,152].

Tissue culture and genetic modification to obtain resistance to factors affecting yield have also been used successfully, especially in the case of resistance to the pantropical insect pest of leguminous crops *Maruca vitrata* (Fabricius, 1787), by creating *Bt* genotypes through the introduction of the *Cry1Ab Bt* gene from *Agrobacterium tumefaciens* [116], as there is no possibility of introducing resistance through a classical method application [153]. Introgression breeding has also been explored with the aim of transferring desirable traits from wild species to cultivated cowpea [116,154].

Recently the investigation of relationships between cultivated plants and insects has increased. The benefits of understanding relationships that govern these systems and finding traits that enhance them are manifold. In terms of crops, the quality and yield of the produced fruits and seeds increases several times, while in terms of pollinating insects, the conservation of their diversity is enhanced and their number is increased, which are major ecological issues due to their rapid decline [155], and the environmental benefits they provide [156,157,158,159]. Morphological and phenological traits of flowers [160], nectar secretion [161] and pollen production [162] as well as the release of volatile aromatic substances [163,164] are key traits to investigate for pollinator attraction enhancement. The ultimate goal is usually to exploit heterosis and create a hybrid resulting in increased yield of a mainly self-pollinated or a partially allogamous crop. Success so far has been observed to be limited in terms of leguminous species [165,166]. However, this could be a solution for cowpea breeding, which is mainly a self-fertilizing species but retains pollinator-attracting features, such as extracellular nectaries [167,168].

The identification of floral traits that are directly related to the prediction of insect-pollinators’ presence and action is very complex [169], with little prediction success [170] and a time-consuming [171] process. The use of molecular markers and genomics could potentially assist towards this breeding approach as well. For example, genetic loci (QTLs) of various aromatic volatiles emitted from cowpea flowers have been reported by Andargie et al. [141], substances that could possibly influence pollinators’ visitation. Therefore, detection or genotyping these genetic loci could probably lead to increased visitation of cowpea genotypes by pollinators.

## 5. Cowpea Plant Genetic Resources for Main Productivity Constraints in Europe

Cowpea faces a multitude of biotic and abiotic factors that negatively affect its productivity worldwide [172]. In Europe, the cowpea cultivation area is increasing [10] as it is considered a drought- and high-temperature-tolerant plant species in comparison to other legumes. Recently, cowpea fresh pods and green seeds started to be investigated as new products for the market [10], while more intense efforts are made to increase pulse production and grain-legume-based products in Europe [173].

Cowpea cultivation in temperate climates lasts from late Spring to early Autumn. Although the area is characterized by mild climatic conditions and the crop is not subjected to such adverse conditions as in Africa [5], cowpea confronts with a plethora of stresses and yield-limiting factors regarding seed and fresh pod production. Exploitation of nutrient content of its fresh pods and seeds is also important worldwide as many substances are essential for humans [174].

Identification of genetic material available and suitable regarding adaptation and tolerance to the main cowpea restrictive factors is the first step for breeding due to the narrow genetic base that characterizes breeding varieties [175]. Wild relatives, exotic germplasm and landraces are sources of hidden diversity. Landraces form the main variability source for cowpea, as there are incompatibility difficulties and production of non-fertile hybrids while inter-crossing with crop wild relatives [45].

According to Genesys PGR [176], a total of 33,832 registered samples of cowpea (*Vigna unguiculata* (L.) Walp.) are stored in gene banks worldwide, with the largest collection held in the International Institute of Tropical Agriculture (IITA) [176]. Regarding their improvement level, the most abundant group consists of landraces with 16,983 entries, while improved varieties amount to 501. Among the accessions that are kept worldwide, 1085 are of European origin, of which most (343) are of Italian origin. Their improvement level is mainly landraces (669), while only sixteen are improved varieties [176]. According to EURISCO [177] 4301 accessions are conserved ex situ in European national gene banks, of which 1508 are kept in the Russian Federation, 498 in Spain, 363 in Italy, 359 in Portugal, 332 in Belgium, 319 in Germany, 310 in Bulgaria, 262 in Hungary, 208 in Romania and 142 in other countries. At the same time, there are few cowpea varieties registered in the national catalogues of European countries [55,178,179,180] compared to the varieties in circulation in the market worldwide, while the species is not mentioned in the Common Catalogues of Varieties of Agricultural Plant and Vegetable Species [181,182].

Cowpea genetic material is also conserved worldwide on-farm through its cultivation by farmers that promote the conservation of landraces, also known as local varieties or local populations, which are most often sown in small-scale plots or gardens (on-farm conservation) for personal use by farmers or to supply the local market in some cases [56]. Cultivated local varieties are usually part of the cuisine and culinary preferences of the region [183,184,185] or accompany some cultivation management tradition [186]. Their cultivation in many cases is so directly intertwined with the local society that they take the name of the region where they are cultivated [20,187,188,189,190]. An advantage of this specific form of conservation is the continuous adaptation to the current soil and climatic conditions and agricultural practices, which allows the material to evolve over time compared to *ex situ* conservation, which is static [191]. On-farm cowpea material conserved therefore constitutes a valuable source of diversity, in most cases underexploited.

### 5.1. Genetic Resources for Abiotic Stress Factors

Cowpea confronts with various abiotic stresses i.e., high temperatures [124], drought especially during flowering and pod setting periods [180,192], photoperiodic requirements [172,193] and soil limiting factors [194] such as salinity and sodicity problems [195]. In Southern Europe, with reduced water availability looming worldwide [14], searching for drought-tolerant genetic material along with production stability under limiting water conditions is of primary importance. The development of drought-tolerant cultivars is based on finding efficient methods to evaluate the tolerance levels of the available germplasm [194], as well as identifying various plant traits [119,195] and physiological and metabolic pathways [196,197] associated with stress resistance.

Due to the short-day photosensitivity that many genotypes present, selection of material appropriate for cultivation in Europe consists also of a breeding goal. Genotypes should be therefore selected depending on the climatic conditions and the duration of the cowpea growing season available in each region. Photosensitive genotypes are late maturing and often do not produce pods until the end of the growing season. As such genotypes are planted during longer day length due to the extended duration of the vegetative stage preventing early transition into reproductive growth, resulting often in higher production [198,199]. Therefore, an appropriate sowing time should be selected for them. Selecting yardlong bean genotypes for photo-insensitivity is also a primary breeding goal [200]. Regarding soil limiting factors, increasing salinity observed in many areas of Southern European countries [201,202] renders this factor important for cowpea production as for other crops. Cowpea also faces problems growing in alkaline soil conditions (pH ≥ 7.5), developing severe leaf chlorosis and stunted plant growth [203].

Crop wild relatives of cowpea were assessed leading to the identification of high levels of abiotic stress tolerance, however efforts proved fruitless due to failure of crossing [204]. Numerous genotypes and accessions, including landraces, have been therefore characterized and evaluated with the goal of being a genetic resource for drought, heat and salinity tolerance [180,205,206,207,208,209,210,211,212,213,214]. Landraces’ potential to tolerate drought and salinity stress under different developmental stages was revealed through these studies (Table 4). Limited screening and evaluation of cowpea landrace material have been done with regard to the rest of abiotic stresses. However, several cowpea genotypes and cultivars have been identified [209,210,215,216], and breeding lines with high temperature tolerance have been developed [49,217]. Landraces and other cultivated material were screened and appeared also to be promising in cultivation under calcareous and high alkaline soils [56,203]. A landrace coded “ID7” was identified as one of the two most suitable genotypes for three different soil types in Sudan [218].

### 5.2. Genetic Resources for Biotic Stress Factors

Pest attacks are the main factors reducing cowpea productivity in many areas worldwide [49,237]. Major insect pests that cause cowpea economic losses include cowpea aphids (*Aphis craccivora* C.L. Koch, 1854), thrips (*Megalurothrips sjostedti* Trybom, 1908), green stink bugs (*Nezara viridula* Linnaeus, 1758) and cowpea weevils (*Callosobruchus maculatus* Fabricius, 1775). In Africa, *Maruca vitrata* (Fabricius, 1787), is one of the most serious pests, causing tremendous losses [238,239], while in Europe, cowpea weevils and aphids constitute the main insect pests. Nematodes also seriously affect cowpea plants worldwide including Europe [240], leading to nutrient deficiencies and stunting or wilting; thus, the root system is incapable of absorbing adequate amounts of water and mineral nutrients. Furthermore, fusarium wilt is also enhanced because of the root injuries caused by nematodes. *Meloidogyne, Pratylenchus* and *Scutellonema* are the most important nematode genera worldwide leading to losses of cowpea yield [241].

The primary goal of several breeding programs worldwide is therefore to find and create varieties with resistance to the main cowpea pests. Many genotypes have been developed and are available in African countries, e.g., TVu-6464, TVu-1583 and TVu-810, from IITA (International Institute of Tropical Agriculture) [242]. However, finding genotypes with resistance to pests in most cases has proved fruitless [49], as it is difficult to find resistant genotypes, and usually their resistance relies on a dominant gene which can be easily overcome [172]. For example, the aphid resistance of the improved line IT97K-499-35 appears to have been overcome by aphid populations in Ghana [243].

A plethora of aphid-resistant improved lines, genotypes and commercial varieties have been identified worldwide [243,244,245,246,247,248], while aphids did not prefer resistant cowpea genotypes and fed significantly less on them [249]. Among the screened genotypes, some landraces and wild cowpea accessions presented resistance to aphids (Table 5). Worth mentioning is that work has also been done in India for biotic stresses resistance [250]. A corresponding investigation has not yet taken place for cowpea genetic material of European origin, however the potential of twelve Greek cowpea landraces to *Aphis craccivora* was highlighted, as they were found to possess the allele CP-171-172 indicative for aphid resistance of the TVu-2876 genotype [59].

In an effort to find genotypes resistant to weevil, both the inability to easily deposit eggs on the seed coat and the reduced rates of hatching adults are taken into account [267]. Based on these factors, Cruz et al. [268] identified four resistant cowpea genotypes. Kalpna et al. [269] reported also that the variety TVu-2027 showed resistance to weevil due to its increased content of trypsin inhibitors and specific amino acids. Lines and genotypes resistant to weevil were also identified [270,271,272]. Recently, Ferreira et al. [173] published the finding of a resistant cultivar (cv. BRS Xiquexique) based on the increased presence of chitin-binding proteins (e.g., chitinases). Wild *Vigna* species such as *V. luteola*, *V. vexillata* and *V. reticulata* are also considered resistant to weevil [273]. Two landraces were also identified by Nyarko et al. [239] to present weevil resistance (Table 5).

Resistance to nematodes of the genus *Meloidogyne* spp. has been found to be a quantitative trait locus based on the abundance of additive genes [274]. Huynh et al. [274] discovered the gene region (QTL) (QRk-vu11.1) harboring the *Rk* gene, which confers resistance or partial resistance to nematodes of the genus *Meloidogyne*. Two more genes, one in a dominant (*Rk^2^*) and one in a recessive form (*rk3*), also contribute complementarily to resistance [275,276]. Varieties CB5, CB27, CB46, CB50 and CB88 in the USA express dominance of this gene [275,277]. The International Institute of Tropical Agriculture (IITA) has also released many nematode-tolerant breeding lines [71,278,279]. Broad-based nematode resistance was reported by Ndeve et al. [257] for cowpea genotypes from South-East Africa, including also landraces. Nematodes resistance was also observed in twelve wild and landrace accessions by Dareus et al. [256] (Table 5).

Fungal diseases such as anthracnose (*Colletotrichum lindemuthianum*), cercospora (*Cercospora canescens*), fusarium (*Fusarium oxysporum* f. sp. *phaseoli*), root rot (*Macrophomina phaseolina*) and septoria (*Septoria vignicola*) infect cowpea worldwide [261,264]. Furthermore, more than twenty different viruses have been recorded that infect cowpea worldwide [53,280,281,282], causing losses ranging from 10 to 100% [283]. Among the most common in Europe are Cowpea-aphid-borne mosaic virus (CABMV), Cowpea mosaic virus (CPMV), Southern bean mosaic virus (SBMV) and Cucumber mosaic virus (CMV) [282].

Several genotypes, including landraces (Table 5), with resistance to cowpea pathogens have been identified, such as for bacterial blight [261], cercospora [284] and fusarium wilt [260]. Diallel crosses were used to breed for resistance to macrophomina root rot disease [285], while eight resistant lines were identified by Lamini et al. [286].

Efforts to find parallel resistance to many pathogens have been underway for years [287,288]. A new variety, VBN 09-013 (VBN 3), was created with parallel resistance to rust, anthracnose and Bean mosaic virus [289]. The genomic regions harboring disease resistance genes are currently beginning to be mapped [290,291,292]. Cultivation of resistant varieties is considered the most effective and environmentally friendly method for confronting viruses diseases. A number of improved resistant lines and varieties to viruses have been released [90,293]. Many resistant landraces along with genotypes and varieties to viruses have also been identified [294,295] (Table 5).

### 5.3. Genetic Resources for Yield Increase and Stability

Cowpea yield is strongly affected by environment (E) and genotype x environment interaction (G × E) [193,296,297,298,299,300] (Table 6). Identification of high-seed-yielding genotypes with broad adaptability and therefore yield stability [301] is considered one of the primary improvement goals [302] in Europe as in the rest of the world. However, cowpea yield is a complex trait, and it is difficult to directly breed for it, as it presents low heritability and pleiotropic effects [303].

Evaluation of genotypes for fresh pod consumption is usually performed separately from seed yield evaluation [5,318,319,320,321], as the desired traits for the consumers are different compared to those for dry seed consumption. For example, the presence of large seed size is not desirable for fresh pod consumption [61], while it is preferable for dry seed production. At the same time, the seeds, after the filling developmental stage (around ten days following their formation), seem to compete with the fresh pods for nutrients such as nitrogen, thus reducing their nutritional value [322].

Nowadays, the observed climatic fluctuations affect yield stability of crops globally [323,324,325]. The ability to produce stable yields in different environments and under changing weather conditions therefore comes to the foreground. Landraces are by definition genetic material that exhibits intermediate yield production capacity but also high yield stability in low-input cropping systems [326,327]. Therefore they can play a major role in finding genotypes that present yield stability [327,328]. The shift towards the stability of performance rather than its maximization has begun to be revealed through participatory breeding programs, and stability is being considered as one of the most desired properties by producers [329].

Numerous landraces have been screened for their seed and fresh pod yield performance under adverse environments [296,330,331,332,333]. Among landraces evaluated, some were proved to yield similarly to improved genotypes, such as the “Akidi Ala” local variety from Nigeria and “Chimponogo” local variety from Zambia [334,335]. Some of them even managed to express higher seed or fresh pod yield compared to the used standard checks [322,336] and presented higher stability [337].

European cowpea landraces have been studied, revealing interesting material regarding their yield potential. In a collection of 48 cowpea accessions evaluated by Stoilova and Pereira [338] including landraces, a landrace from Bulgaria named “A4E007” was among the genotypes selected to be included in a breeding programme aiming to increase seed yield. Martos-Fuentes et al. [193], evaluating, at three locations and two consecutive years, twelve cowpea genotypes, consisting mainly of landraces material from Greece, Portugal and Spain, defined cowpea landraces with high productivity levels such as “BGE038474” in the Cartagena region and “Vg73” at the Elvas site. In Vila Real the most promising landraces were “Cp5553” and “Vg60” from Portugal and “AUA1” from Greece, depending on the experimental year [193]. Statistically significant differences were observed in a Greek cowpea collection assessed by Lazaridi et al. [56] regarding their traits related to yield, revealing useful breeding material. Among the landraces, “VG18”produced the highest number of pods per plant, while “VG19” presented the highest seed weight per plant.

Cowpea fresh pods are also consumed as vegetables in many countries in Europe; in this direction some European cowpea landraces have also been studied. Lazaridi et al. [332] preliminary evaluating cowpea landraces from Southern European countries found that the “Cp5128” landrace from Portugal resulted in a higher number of fresh pods per plant and fresh pod weight per plant in comparison to the other landraces, providing therefore valuable material for cultivation and breeding purposes regarding fresh pod production. Omirou et al. [339] managed to increase cowpea fresh pod yield of the “Argaka” landrace, a traditional landrace of Cyprus, through whole plant phenotyping and continuous selections. Pekşen and Pekşen [320] evaluating twelve breeding lines developed from landraces, defined new material (L3, L12, L13 lines) that statistically significantly exceeded fresh pod yield of cowpea varieties that were included in the experiment.

### 5.4. Genetic Resources for Enhanced Nutritional Value

Cowpea nutritional value and its importance is widely reported [2,340]. Recently, the species has gained more attention due to its positive effects on human health [6,341]. Food dishes containing cowpea leaves especially are characterized by extremely high Fe and Ca levels [342]. Recently, in Europe the consumption of high protein vegetables, such as cowpea, is intensively promoted [10]. Cowpea is characterized by high seed protein content even higher than 30% [343,344] and low-fat content as well as a significant concentration of carbohydrates [3,345].

Padhi et al. [345], evaluating 120 cowpea genotypes, found remarkable variability among them regarding their proximate composition. Proximate and nutrient composition of cowpea fresh pods and seeds have been found to be strongly affected by environment [15] and genotype [346,347,348], while a genotype x environment interaction is also usually present [10,174,349]. Cooking methods and food processing are also reported to drastically affect cowpea nutrient content [3,350], e.g., fermentation of cowpea flour was found to improve P availability and Mg digestibility [351]. Recently, cowpea flour up to 4% was incorporated successfully in a traditional plate called “Kırklareli meatballs” in Turkey, aiming to improve its nutritional content without changing its textural and sensory properties [13].

Cowpea is rich in proteins and carbohydrates but its essential micronutrients content is not satisfactory. Biofortification of cowpea is considered crucial to target for an adequate dietary intake of micronutrients [352,353]. Strong correlations among seed crude protein and Fe and Zn contents [354] and between K and Mg as well as between Ca and Mg [56] indicate the possibility of breeding for increased concentrations of some nutrient components simultaneously. However, cowpea contains various amounts of several antinutritional factors such as alkaloids, flavonoids, saponins, tannins [355], phenols and phytic acid [345] in seeds, and oxalates, phytates and nitrates in leaves, reducing its nutritional value [356]. Statistically significant differences were observed among European cowpea cultivars regarding their antinutritional factors [357]. Karapanos et al. [61], evaluating the fresh pods of 36 cowpea landraces and a cowpea variety from Southern Europe for nutritional value, recorded low raffinose-family oligosaccharides (RFOs) and nitrate concentrations similar to those of snap bean pods and lower than leafy vegetables.

Several studies aimed to identify cowpea accessions with high nutrient content in anticipation of alleviating malnutrition [352] and micronutrients deficiencies observed in low- and middle-income countries [358], as well as to promote the creation of new vegetable products for the market [10,61]. Among the accessions, some landraces were also included that often exhibited desirable proximate and nutrient values.

Proximate composition of cowpea seeds, cultivated in Bulgaria, ranged from 22.5 to 25.6% protein content, 28.3 to 36.2% starch, 1.3 to 1.9% fat, 1.7 to 3.0% insoluble fiber and 3.2 to 3.7% minerals [346]. Among the accessions evaluated, two Bulgarian landraces presented higher protein and starch seed content than the ex situ obtained material. Their oil, sterol, tocopherol and phospholipid content was also equal or higher than the rest of the accessions studied [344]. Verma et al. [359] found that the Indian landrace “Pusa Komal” had significantly lower proximate seed composition in comparison to seven improved varieties, while in Swaziland a local variety “Mtilane” also compared with five improved lines and presented no statistically significant differences for seed crude protein and fat content [360]. Its nutrient seed content (Ca, Fe, Zn) was also equivalent to the assessed breeding lines [360]. High Fe seed content (67 μg g^−1^) was recorded in the “Soronko” landrace, while a very high seed protein content, up to 40%, was recorded in the “Bengpla” landrace [15]. The “Bengpla” landrace along with the “Glenda” landrace also exhibited extremely high leaf protein content [15]. Significant statistical differences were recorded for seven cowpea landraces from Indonesia that were compared with three commercial varieties. Among them, landrace “KM7” presented high lipid and protein content, and “KM1” presented high carbohydrate and folic acid content [361]. A local variety “GH3684” presented lower carbohydrate seed content but higher fiber content compared to six advanced and parental lines [362].

Cowpea landraces with European origin have been evaluated regarding the proximate and nutrient composition of their seeds, fresh seeds and green pods, revealing some promising genotypes such as “Cp5647” and “Cp4877” originating from Portugal that exhibited high total soluble solids (7.6 and 6.5 °Brix, respectively) and titratable acidity of fresh pods [61], “BGE038477” and “BGE038478” from Spain that presented high seed antioxidant capacity (17.78 mg GA g^−1^ dw and 18.26 mg GA g^−1^ dw, respectively) and phenolic content [10], “AUA2” from Greece that presented high Ca (6.10 g kg^−1^), Mg (3.40 g kg^−1^), S (1.21 g kg^−1^), B (20.60 mg kg^−1^) and Zn (64.10 mg kg^−1^) content of fresh pods and P (5.40 g kg^−1^) and Fe (70.90 mg kg^−1^) content of immature seeds [10] as well as “BGE038477”, “BGE038478” and “VG20” that presented high seed protein content, 29.52%, 29.46% and 28.37%, respectively [10,56].

## 6. Conclusions

Cowpea, an orphan legume and a staple crop for African countries, has gained breeders’ attention in recent years worldwide. Southern European countries traditionally cultivate cowpea, using as the main cultivating material landraces, and thus a hidden genetic diversity has been conserved. Cowpea landraces have been proved valuable sources of tolerance to abiotic stresses and of resistance to main pests and pathogens prevailing worldwide. However, the majority of cowpea plant genetic resources remain unexploited. Cowpea landraces originating from Europe could provide valuable material for breeding, combining tolerance and resistance characteristics but also adaptability to the climatic and soil conditions of the area. Despite the progress that has been made recently, through the implementation of classical and modern breeding techniques, more research is necessary to further access their potential, to develop high yielding varieties and to create new variable and nutritional products for the market.

## Figures and Tables

**Table 1:** Heritability in the broad and narrow sense, and genetic advance of cowpea (*Vigna unguiculata* cv-gr unguiculata) traits.

Trait	Broad Sense Heritability (*H*) (%)	Narrow Sense Heritability (*h^2^*) (%)	Genetic Advance	References
Days to flowering	83.22		4.48	Araméndiz-Tatis et al. [74]
	97.17		18.34	Belay and Fisseha [75]
	61.32		3.81	Devi and Jayamani [76]
	71.25		9.45	Diriba et al. [77]
	63.77	50.11		dos Santos et al. [78]
	89.93		6.74	Jonah and Fakuta [79]
	41.92			Inuwa et al. [80]
		63.20–86.00		Ishiyaku et al. [81]
	96.74			Manggoel et al. [82]
	91.93			Mofokeng et al. [83]
	84.78			Omoigui et al. [84]
	57.84	42.16		Owusu et al. [85]
	77.72		5.56	Owusu et al. [86]
	62.00–85.00	18.00–85.00	1.32–7.53	Owusu et al. [87]
	37.46–86.43	4.26–82.34	8.91–33.51	Pathak et al. [88]
	50.00			Shimelis and Shiringani [89]
	86.00			Singh et al. [90]
	87.72		11.60	Vavilapalli et al. [91]
Days to maturity	85.57		11.37	Belay and Fisseha [75]
	73.54		7.50	Devi and Jayamani [76]
	75.40		13.04	Diriba et al. [77]
	71.85	53.97		dos Santos et al. [78]
	30.45			Inuwa et al. [80]
	79.98			Omoigui et al. [84]
	73.03	26.97		Owusu et al. [85]
	89.58		6.58	Owusu et al. [86]
	72.00–84.00	8.00–62.00	0.89–8.93	Owusu et al. [87]
	34.52–95.83	4.79–93.73		Pathak et al. [88]
	66.00			Shimelis and Shiringani [89]
	82.00			Singh et al. [90]
	92.58		8.68	Vavilapalli et al. [91]
Plant height	98.78		28.10	Belay and Fisseha [75]
	60.54		13.19	Devi and Jayamani [76]
	88.58			Omoigui et al. [84]
	78.51		14.27	Owusu et al. [86]
	97.42		64.32	Vavilapalli et al. [91]
Pod length	85.11		4.25	Belay and Fisseha [75]
	75.86		3.34	Devi and Jayamani [76]
	80.64		4.32	Diriba et al. [77]
	41.63		5.35	Jonah and Fakuta [79]
	63.16			Manggoel et al. [82]
	42.94			Omoigui et al. [84]
	98.38		48.70	Vavilapalli et al. [91]
	94.86	5.14		Ayo-Vaughan et al. [92]
Number of seeds per pod	68.01		2.20	Belay and Fisseha [75]
	48.51		1.63	Devi and Jayamani [76]
	55.97		2.28	Diriba et al. [77]
	90.29		11.29	Jonah and Fakuta [79]
	21.15			Inuwa et al. [80]
	73.40			Manggoel et al. [82]
	96.21			Mofokeng et al. [83]
	75.82			Omoigui et al. [84]
	55.03		2.11	Owusu et al. [86]
	75.00			Singh et al. [90]
	98.96	1.04		Ayo-Vaughan et al. [92]
	20.80–81.50			Drabo et al. [93]
Number of pods per plant	54.92		2.77	Araméndiz-Tatis et al. [74]
	80.34		5.35	Devi and Jayamani [76]
	35.09		4.52	Diriba et al. [77]
	51.70		15.37	Jonah and Fakuta [79]
	89.23			Manggoel et al. [82]
	91.46			Mofokeng et al. [83]
	19.25			Omoigui et al. [84]
	23.00			Shimelis and Shiringani [89]
	85.00			Singh et al. [90]
Hundred/thousand seed weight	97.99		8.89	Araméndiz-Tatis et al. [74]
	94.30		4.43	Devi and Jayamani [76]
	84.41		5.19	Diriba et al. [77]
	91.48		15.21	Jonah and Fakuta [79]
	86.84			Manggoel et al. [82]
	97.15			Mofokeng et al. [83]
	96.19			Omoigui et al. [84]
	91.52		4.40	Owusu et al. [86]
	11.00			Shimelis and Shiringani [89]
	91.00			Singh et al. [90]
	98.99	1.01		Ayo-Vaughan et al. [92]
Seed size	48.00–90.20			Drabo et al. [93]
Seed yield	65.32		8.25	Araméndiz-Tatis et al. [74]
	67.78		4.55	Devi and Jayamani [76]
	62.39		21.27	Jonah and Fakuta [79]
	90.91			Manggoel et al. [82]
	38.36			Mofokeng et al. [83]
	51.68			Omoigui et al. [84]
	55.00			Shimelis and Shiringani [89]
	83.00			Singh et al. [90]

**Table 2:** Heritability in the broad sense and genetic advance of cowpea (*Vigna unguiculata* cv-gr sesquipedalis) traits.

Trait	Broad Sense Heritability (*H*) (%)	Genetic Advance	References
Days to flowering	83.00	3.89	Rambabu et al. [94]
	41.00	1.60	Bhagavati et al. [95]
	96.13	17.75	Haque et al. [96]
	29.50		Kongjaimun et al. [97]
	71.73	17.17	Lovely and Radahavedi [98]
	55.33	2.72	Sharma et al. [99]
	76.88	2.62	Sultana et al. [100]
	80.40	12.36	Vinay et al. [101]
Days to maturity	24.00	1.89	Rambabu et al. [94]
	44.00	2.04	Bhagavati et al. [95]
	47.20		Kongjaimun et al. [97]
	43.38	5.35	Lovely and Radahavedi [98]
	88.90	2.45	Sharma et al. [99]
	73.00	6.70	Vinay et al. [101]
Plant height	79.00	87.73	Rambabu et al. [94]
	84.00	100.7	Bhagavati et al. [95]
	91.13	19.73	Haque et al. [96]
	82.30	39.89	Lovely and Radahavedi [98]
	99.53	65.40	Sharma et al. [99]
	93.30	44.18	Vinay et al. [101]
Pod dehiscence	99.90		Kongjaimun et al. [97]
Pod length	99.00	25.39	Rambabu et al. [94]
	95.00	23.44	Bhagavati et al. [95]
	90.83	18.04	Haque et al. [96]
	98.21	66.76	Lovely and Radahavedi [98]
	57.10	9.00	Sultana et al. [100]
	85.30	30.88	Vinay et al. [101]
	75.00		Amusa et al. [102]
	47.70		Kusmiyati et al. [103]
	85.81		Ramkumar and Anuja [104]
Number of seeds per pod	62.00	2.21	Rambabu et al. [94]
	26.00	0.92	Bhagavati et al. [95]
	81.50	5.73	Haque et al. [96]
	69.70–95.70		Kongjaimun et al. [97]
	42.20	1.04	Sultana et al. [100]
	73.50	31.16	Vinay et al. [101]
Hundred/thousand seed weight	96.00	5.38	Rambabu et al. [94]
	93.00	6.91	Bhagavati et al. [95]
	97.67	5.86	Haque et al. [96]
	91.21	2.83	Sultana et al. [100]
	85.10	51.8	Vinay et al. [101]
	96.00		Amusa et al. [102]
Number of seeds per plant	23.00		Kusmiyati et al. [103]
Number of pods per plant	83.00	53.11	Rambabu et al. [94]
	66.00	49.42	Bhagavati et al. [95]
	85.60		Kongjaimun et al. [97]
	76.31	80.88	Lovely and Radahavedi [98]
	85.90	4.84	Sharma et al. [99]
	96.78	4.12	Sultana et al. [100]
	43.10		Kusmiyati et al. [103]
	30.04		Ramkumar and Anuja [104]
	64.00		Umaharan et al. [105]
Fresh pod yield	75.00	509.60	Rambabu et al. [94]
	91.00	4.27	Bhagavati et al. [95]
	98.76	136.99	Haque et al. [96]
	77.00	76.44	Lovely and Radahavedi [98]
	94.60	32.60	Sharma et al. [99]
	90.01	58.95	Sultana et al. [100]
	96.18		Ramkumar and Anuja [104]
Seed yield	27.80–81.80		Kongjaimun et al. [97]

**Table 3:** Indicative Quantitative trait loci (QTLs) associated with cowpea (*Vigna unguiculata* (L.) Walp.) yield and linkage groups to which they belong.

Trait	Linkage Groups	Number of QTLs	QTLs	References
Days to flowering	Vu05, Vu09	2	*CFt5, CFt9*	Lo et al. [115]
	Vu01, Vu02, Vu07	3	*qdf1, qdf2, qdf7*	Andargie et al. [126]
	LG1, LG2, LG4, LG5, LG6, LG7, LG8, LG9, LG10, LG11	10	*Fld1.1, Fld2.1, Fld4.1, Fld5.1, Fld6.1, Fld7.1, Fld8.1, Fld9.1, Fld10.1, Fld11.1*	Kongjaimun et al. [127]
	Chr2, Chr9	2	*qdtf2.1, qdtf9.1*	Angira et al. [128]
Peduncle length	Vu05	1	*CPedl5*	Lo et al. [129]
	LG1, LG7, LG10	3	*qPeL1, qPeL8, qPeL10*	Garcia-Oliveira et al. [130]
Number of inflorescences per plant	LG2,LG9	3	*qPeN2.1, qPeN2.2, qPeN9*	Garcia-Oliveira et al. [130]
Days to maturity	LG1, LG2, LG3, LG4, LG6, LG7	6	*Pddm1.1, Pddm2.1, Pddm3.1, Pddm4.1, Pddm6.1, Pddm7.1*	Kongjaimun et al. [127]
Growth habit	Vu01	1	*qgh* (in the same position with *qsw1*)	Andargie et al. [126]
Plant height	Chr4, Chr9	3	*qPH4.1,qPH4.2, qPH9.1*	Angira et al. [128]
Pod dehiscence	Vu05	1	*qps5* (in the same position with *qpft5*)	Andargie et al. [126]
	LG1, LG4, LG7, LG9	4	*Pdt1.1, Pdt4.1, Pdt7.1, Pdt9.1*	Kongjaimun et al. [127]
	Vu03, Vu05	2	*CPshat3, CPshat5*	Lo et al. [129]
	Vu07	1	*Shat7.1.1*	Watcharatpong et al. [131]
Pod length	LG1, LG2, LG3, LG4, LG5, LG7, LG8, LG9	8	*Pdl1.1, Pdl2.1, Pdl3.1, Pdl4.1, Pdl5.1, Pdl7.1, Pdl8.1, Pdl9.1*	Kongjaimun et al. [127]
	Vu03, Vu08	2	*CPodl3, CPodl8*	Lo et al. [129]
	LG3, LG4, LG5, LG7, LG8, LG10	6	*qPoL3, qPoL4, qPoL5, qPoL7, qPoL8, qPoL10*	Garcia-Oliveira et al. [130]
	LG4, LG6, LG9, LG11	4	*Qcpl-1, Qcpl-2, Qcpl-3, Qcpl-4*	Pan et al. [132]
	LG3, LG5	2	*Qpl.zaas-3, Qpl.zaas-5*	Xu et al. [133]
Number of pods per plant	LG8	1	*qPoN8*	Garcia-Oliveira et al. [130]
Number of seeds per pod	LG7, LG11	2	*Sdnppd7.1, Sdnppd11.1*	Kongjaimun et al. [127]
	Vu05, Vu09	2	*CSp5, CSp9*	Lo et al. [129]
	LG8, LG9, LG11	4	*qSN8, qSN9.1, qSN9.2, qSN911*	Garcia-Oliveira et al. [130]
	LG5, LG11	2	*Qcgn-1, Qcgn-2*	Pan et al. [132]
Hundred seed weight	LG1, LG3, LG4, LG5, LG6, LG7, LG8, LG10	8	*Sd100wt1.1, Sd100wt1.2, Sd100wt3.1, Sd100wt4.1, Sd100wt5.1, Sd100wt6.1, Sd100wt7.1, Sd100wt8.1, Sd100wt10.1, Sd100wt11.1*	Kongjaimun et al. [127]
	Vu01, Vu06, Vu08	3	*CSw1, CSw6, CSw8*	Lo et al. [129]
	LG7, LG8, LG9	3	*qSW7, qSW8, qSW9*	Garcia-Oliveira et al. [130]
	LG4, LG7, LG10, LG11	4	*Qctgw-1, Qctgw-2, Qctgw-3, Qctgw-4*	Pan et al. [132]
Seed weight per plant	Vu01, Vu02, Vu03, Vu07, Vu10	7	*qsw1, qsw2.1, qsw2.2, qsw3.1, qsw3.2,qsw7, qsw10*	Andargie et al. [126]
	LG2, LG4, LG5, LG6, LG7, LG10	6	*Sdtwt2.1, Sdtwt4.1, Sdtwt5.1, Sdtwt6.1, Sdtwt7.1, Sdtwt10.1*	Kongjaimun et al. [127]
	Vu01, Vu02, Vu03, Vu10	6	*qsw1, qsw2.1, qsw2.2, qsw3.1, qsw3.2, qsw10*	Andargie et al. [133]
Seed yield	LG1, LG3, LG4, LG7, G8, LG11	6	*Dro-1, Dro-3, Dro-4, Dro-7, Dro-8, Dro-10*	Muchero et al. [123]

**Table 4:** Cowpea landraces with potential to tolerate drought and salinity stress.

Abiotic Stresses	Landraces	Developmental Stage That Stress Was Studied	References
Drought stress	NLLP-CPC-07-145-21, NLLP-CPC-103-B, NLLP_CPC-07-54	Throughout life cycle, yield	Mekonnen et al. [5]
	L1, L3	Early reproductive stage, yield	Nunes et al. [180]
	Gorom local	Vegetative stage	Hamidou et al. [194]
	DWDCC001, DWDCC006, DWDCC015	Throughout life cycle, yield	Hedge and Mishra [205]
	KM7	Throughout life cycle	Karuwal et al. [219]
	C1, C18, C44, C47, C50, C54	Germination stage	Carvalho et al. [220]
	Kanannado, Aloka local	Seedling stage	Nkomo et al. [221]
	A116	Vegetative stage	Gomes et al. [222]
	Timbawene moteado	Vegetative stage	Martins et al. [223]
	AUALIMNO133	Throughout life cycle, yield	Andreopoulou [224]
	C11, C47, C56	Throughout life cycle, yield	Santos et al. [225]
	Menia	Vegetative stage	Zegaoui et al. [226]
	C13, C25	Trifoliate stage till pod formation stage, yield	Gull et al. [227]
	Kpodjiguegue	Vegetative and reproductive stages	Ezin et al. [228]
	TVu-4886	Flowering stage	Ajayi et al. [229]
	PI 527263, PI 5272302	Throughout life cycle, yield	Yahaya et al. [230]
	Zhemchyuzhina Kaspiya, Astrakhanskaya krasavitsa, Kaspiyskaya zarya	Throughout life cycle, yield	Burlyaeva et al. [231]
Salinity stress	Zhemchyuzhina Kaspiya, Astrakhanskaya krasavitsa, Kaspiyskaya zarya	Throughout life cycle, yield	Burlyaeva et al. [231]
	Sesenteño	Vegetative stage	Murillo-Amador et al. [232]
	EK1, TZ7, B23	Germination stage	Nabi et al. [233]
	PI 582570	Germination stage	Ravelombola et al. [234]
	58–78, 58–191	Germination stage	Thiam et al. [235]
	Siyah Sürmeli, Serodor Cambados, Acebek	Early vegetative stage	Daşgan et al. [236]

**Table 5:** Wild *Vigna* material and cowpea landraces with resistance or immunity to main pests and diseases/pathogens prevailing in Europe.

Pests	Wild *Vigna*/Cowpea Landraces	References
Aphids	UCR779 (PI 583014)	Muchero et al. [123]
	TVNu 1158	Souleymane et al. [245]
	Golam White	Omoigui et al. [246]
	B261-B, B383	Machacha et al. [251]
	Enrica Pobre (CE-36), Das Almas (CE-07), CE-51 (selected within CE-13), Ritinha (CE-08)	De Lima Fereira et al. [252]
	Mandya local	Jayappa and Lingappa [253]
Thrips	Sanzisabinli	Boukar et al.; Togola et al. [254,255]
Weevil	Adom, Bengpla	Nyarko et al. [239]
Nematodes	TVu-12897, TVu-16220	Dareus et al. [256]
	Gile-K-local	Ndeve et al. [257]
Diseases/Pathogens		
Anthracnose	Arimbra Local Kanakamony, Anaswara, Bagyalakshmi, Arka Samradhi	Shiny et al. [258] Merin et al. [259]
*Fusarium redolens*	WC67A, WC27	Namasaka et al. [260]
Bacterial blight	TVu58, TVu64, TVu102, TVu41, TVu87, TVu52	Durojaye et al. [261]
	TVu-5549, TVu-12349	Okechukwu and Ekpo [262]
*Eryshiphe polygoni*	ZN016	Wu et al. [263]
Viruses	WC32, WC18, NE43, NE15, WC35B	Mbeyagala et al. [264]
	Trang 1, Taitar	Milosevic [265]
	C14, C24, C36	Sofi et al. [266]

**Table 6:** Significance of genotypic effect (G), environmental effect (E) and genotype x environment interaction (G × E) while evaluating cowpea genetic material.

Number of Genotypes Evaluated	Number of Cultivation Years/Seasons	Number of Locations	G	E	G × E	References
100	1	1	*p* ≤ 0.001	*p* ≤ 0.001	n.s.	Mofokeng et al. [83]
17	3	1	*p* ≤ 0.001	*p* ≤ 0.001	*p* ≤ 0.001	Owusu et al. [86]
12	2	3	*p* ≤ 0.001	*p* ≤ 0.001	*p* ≤ 0.001	Martos-Fuentes et al. [193]
75	2	3	*p* ≤ 0.001	*p* ≤ 0.001	*p* ≤ 0.001	Mbuma et al. [297]
20	2	3	*p* ≤ 0.01	*p* ≤ 0.01	*p* ≤ 0.01	Goa et al. [299]
50	2	5	*p* ≤ 0.01	*p* ≤ 0.01	*p* ≤ 0.01	Gumede et al. [300]
36	3	3	*p* ≤ 0.001	*p* ≤ 0.001	*p* ≤ 0.001	Abiriga et al. [303]
11	2	1	*p* ≤ 0.05	*p* ≤ 0.001	n.s.	Adewale et al. [304]
25	2	1	*p* ≤ 0.01	*p* ≤ 0.01	*p* ≤ 0.01	Ajayi and Gbadamosi [305]
21	2	1	*p* ≤ 0.001	*p* ≤ 0.001	*p* ≤ 0.01	Aliyu and Makinde [306]
11	1	3	*p* ≤ 0.01	*p* ≤ 0.05	*p* ≤ 0.01	Aliyu et al. [307]
28	2	3	*p* ≤ 0.05	*p* ≤ 0.05	*p* ≤ 0.05	Ddamulira et al. [308]
12	2	4	*p* ≤ 0.01	*p* ≤ 0.01	*p* ≤ 0.01	de Souza Tomaz et al. [309]
15	2	3	*p* ≤ 0.01	*p* ≤ 0.001	*p* ≤ 0.05	Gerrano et al. [310]
37	2	3	*p* ≤ 0.001	*p* ≤ 0.001	*p* ≤ 0.001	Horn et al. [311]
12	2	3	*p* ≤ 0.001	*p* ≤ 0.001	*p* ≤ 0.001	Kuruma et al. [312]
12	1	3	*p* ≤ 0.001	*p* ≤ 0.001	*p* ≤ 0.05	Mukendi et al. [313]
20	2	3	*p* ≤ 0.01	*p* ≤ 0.01	*p* ≤ 0.01	Nunes et al. [314]
20	2	2	*p* ≤ 0.01	n.s.*	*p* ≤ 0.01	Olayemi Odeseye et al. [315]
8	2	5	*p* ≤ 0.01	*p* ≤ 0.01	*p* ≤ 0.01	Owusu et al. [316]
20	2	4	*p* ≤ 0.01	*p* ≤ 0.01	*p* ≤ 0.01	Santos et al. [317]

* n.s.: non-significant.

## Data Availability

Not applicable.
